# Financing for tuberculosis prevention, diagnosis and treatment services in the Western Pacific Region in 2005–2020

**DOI:** 10.5365/wpsar.2023.14.3.976

**Published:** 2023-08-18

**Authors:** Fukushi Morishita, Hend Elsayed, Tauhid Islam, Kalpeshsinh Rahevar, Kyung Hyun Oh, Manami Yanagawa, Katherine Floyd, Inés Garcia Baena

**Affiliations:** a End TB and Leprosy Unit, World Health Organization Regional Office for the Western Pacific, Manila, Philippines.; b Global TB Programme, World Health Organization, Geneva, Switzerland.

## Abstract

**Objective:**

This paper provides an overview of financing for tuberculosis (TB) prevention, diagnostic and treatment services in the World Health Organization (WHO) Western Pacific Region during 2005–2020.

**Methods:**

This analysis uses the WHO global TB finance database to describe TB funding during 2005–2020 in 18 low- and middle-income countries (LMICs) in the Western Pacific Region, with additional country-level data and analysis for seven priority countries: Cambodia, China, the Lao People's Democratic Republic, Mongolia, Papua New Guinea, the Philippines and Viet Nam.

**Results:**

Funding for the provision of TB prevention, diagnostic and treatment services in the 18 LMICs tripled from

US$ 358 million in 2005 to US$ 1061 million in 2020, driven largely by increases in domestic funding, which rose from US$ 325 million to US$ 939 million over the same period. In the seven priority countries, TB investments also tripled, from US$ 340 million in 2005 to US$ 1020 million in 2020. China alone accounted for much of this growth, increasing its financing for TB programmes and services fivefold, from US$ 160 million to US$ 784 million. The latest country forecasts estimate that US$ 3.8 billion will be required to fight TB in the seven priority countries by 2025, which means that unless additional funding is mobilized, the funding gap will increase from US$ 326 million in 2020 to US$ 830 million by 2025.

**Discussion:**

Increases in domestic funding over the past 15 years reflect a firm political commitment to ending TB. However, current funding levels do not meet the required needs to finance the national TB strategic plans in the priority countries. An urgent step-up of public financing efforts is required to reduce the burden of TB in the Western Pacific Region.

Tuberculosis (TB) remains a major public health concern, responsible for 10.6 million people falling ill and 1.6 million (including 187 000 people with HIV) dying globally in 2021. (*1*) Worldwide, TB is the 13th leading cause of death and in 2021 was the second leading infectious killer after coronavirus disease (COVID-19). Currently, TB kills more people than HIV-related disease and is a major contributor to antimicrobial resistance-related deaths. (*1*) Despite a steady decline in the TB disease burden over the past two decades, progress towards the target of reducing TB deaths by 90% from 2015 levels by 2030 – a target set by the World Health Organization (WHO) End TB Strategy and the United Nations (United Nations) Sustainable Development Goals (SDGs) – has been slow. Furthermore, the COVID-19 pandemic has reversed years of progress in providing TB services and reducing the TB disease burden.

Since 2002, WHO has been monitoring the funding of TB prevention, diagnostic and treatment services, based on data supplied by national TB programmes (NTPs), as part of its annual rounds of global TB data collection. Findings have been published in global TB reports and in the scientific literature. The latest annual rounds of data collection reflect the evolution in global TB strategies and capture investments in the implementation of new drug regimens, provider-initiated screening, novel diagnostics and the introduction of digital tools for surveillance and case management. These most recent data on global TB spending have revealed changes in the current TB financing landscape, shaped largely by shifts in government and external donor agency priorities at the global and regional levels.

Adequate and sustainable financing is essential for achieving universal health coverage, including access to quality TB services. Given the ambitious targets to end TB by 2030, levels of domestic and international funding for TB response based on historical allocations are now no longer adequate to meet the investments required by current global and country response plans. (*2*) Recognizing this shortfall and the need to accelerate the investment response, in 2018 world leaders attending the first United Nations High-Level Meeting (UNHLM) on TB committed to mobilizing US$ 13 billion a year to finance TB prevention, diagnosis and treatment by 2022 and US$ 2 billion a year to support TB research. (*3*) It is anticipated that world leaders will renew their commitment to accelerate the investment response to end TB at the second UNHLM on TB, scheduled to take place in 2023. According to WHO estimates, in 2020, with the COVID-19 pandemic altering access and delivery models for TB services, funding availed for TB prevention, diagnosis and treatment in 136 low- and middle-income countries (LMICs) totalled US$ 5.3 billion, less than half (41%) of the annual investments required to fund the global TB response to meet End TB targets. At US$ 901 million in 2019, funding for TB research also fell short of annual global investment targets (45%). (*1*)

The WHO Western Pacific Region is home to a quarter of the world’s population (1.9 billion people) across 37 countries and areas, including 18 LMICs. In 2020, the Western Pacific Region accounted for 18% of the estimated global TB incidence, 19% of globally reported TB cases and 6% of estimated global TB deaths. (*1*, *4*) This demographically diverse region comprises both large countries with populations exceeding 1 billion people and small Pacific island countries and areas with just a few thousand residents. The Region is also diverse in terms of the TB burden, with some countries in the pre-elimination stage (defined as < 10 TB cases per 1 million people) while others have high or intermediate TB burdens. (*5*) China, Mongolia, Papua New Guinea, the Philippines and Viet Nam are on the current list of 30 high-TB burden countries (2021–2025), and Cambodia is on the global TB watchlist. (*1*) Kiribati, the Lao People's Democratic Republic, Malaysia and the Marshall Islands are also among the 10 countries classified as regional priority countries for TB in the Western Pacific Region. (*2*)

In line with the global End TB Strategy, the *Western Pacific Regional Framework to End TB: 2021–2030* reaffirmed the importance of adequate financing for TB, effective financial management, and transition from external to domestic funding through strong political commitment and accountability. (*2*) To provide background to the regional TB framework and to inform its investment forecasts, this paper describes historic trends in financing for TB prevention, diagnostic and treatment services in the Western Pacific Region for the period 2005–2020, focusing on 18 LMICs that together accounted for 96% of the Region’s notified cases in 2020. In addition, it presents estimated TB funding requirements for 2021–2025 for seven TB priority countries, based on information reported in their national strategic plans (NSPs). The seven priority countries are Cambodia, China, the Lao People's Democratic Republic, Mongolia, Papua New Guinea, the Philippines and Viet Nam.

## Methods

This descriptive analysis used data from the WHO global TB finance database, as of October 2021. This database contains data on yearly budgets, expenditure and use of TB health services, (*4*) information that countries and areas are required to report annually via WHO’s global TB data collection system. (*6*) It also includes data on spending on inpatient and outpatient care, as estimated by WHO. (*1*)

This analysis distinguishes two major sources of TB financing: domestic and international donor funding. Total domestic funding for tuberculosis captures (1) “domestic” funding, which is funding for selected national TB programmes as reported by countries to WHO, as well as (2) “estimated domestic funds” for inpatient and outpatient care, which is estimated by WHO. International funding includes donor-funded TB investments as reported by national TB programmes to WHO. The methods used to estimate TB funding and costs are described in greater detail elsewhere. (*7*)

For the group of 18 LMICs in the Western Pacific Region, data from the WHO TB finance database were used to review trends in TB funding, disaggregated by funding source and by category of expenditure over the period 2005–2020. (*7*) For the seven priority countries, country-level funding data for 2005–2020 were examined, overall and by funding source, and by category of expenditure for 2020. For the seven priority countries, the analysis also included comparisons of the amount of domestic funding (as a proportion of total TB funding) between 2015 and 2020 and implementation rates (the proportion of spent funds to received funds) during 2015–2020. Country-reported TB budget requirements, forecasted funding gaps (i.e. the difference between the budget required and the expected funding for subsequent years at the time of reporting) and actual funding gaps (i.e. the difference between the budget required and the actual received funding) were also calculated for each year during 2005–2020. Projected funding gaps for 2021–2025 were calculated from required annual budgets provided in NSPs; for the three countries where this information was not available, the budget required was projected using the average annual change in required funding for previous years (for China, this was for 2015–2020, for Mongolia, 2019–2023 and for the Philippines, 2024–2025). Anticipated availability of domestic and international funds to meet the NSP needs for 2021–2025 were estimated using the average annual change in received funding for 2021–2025 and assuming recent patterns would continue. Finally, we estimated the impact of the COVID-19 pandemic on TB financing in 2020 using country-reported information on re-allocation of TB budgets to the COVID-19 response. This information was collected through an online WHO survey added to 2020s annual TB data collection round, complemented by – in the case of the Philippines – data from the Philippine Department of Health.

Data analyses and visualizations were performed using the statistical software package R 3.6.2 (Comprehensive R Archive Network: https://cran.r-project.org/).

## Results

### TB financing patterns in 18 LMICs

Total funding for TB prevention, diagnostic and treatment services in the group of 18 LMICs in the Western Pacific Region more than tripled in 15 years, from US$ 358 million in 2005 to US$ 1061 million in 2020 (**Fig. 1A**). Since 2015, when the End TB Strategy was launched, total TB funding increased by 1.4 times, largely driven by a rise in domestic funding, which increased from US$ 325 to US$ 939 million between 2005 and 2020. International funding for TB response in the Western Pacific Region increased from US$ 33 million in 2005 to a peak of US$ 185 million in 2013 before falling back to US$ 122 million in 2020. Throughout this 15-year period, the Global Fund to Fight AIDS, Tuberculosis and Malaria has provided a substantial proportion of the international funds; in 2020, 81% of the donor funding came from the Global Fund.

**Fig. 1a:**
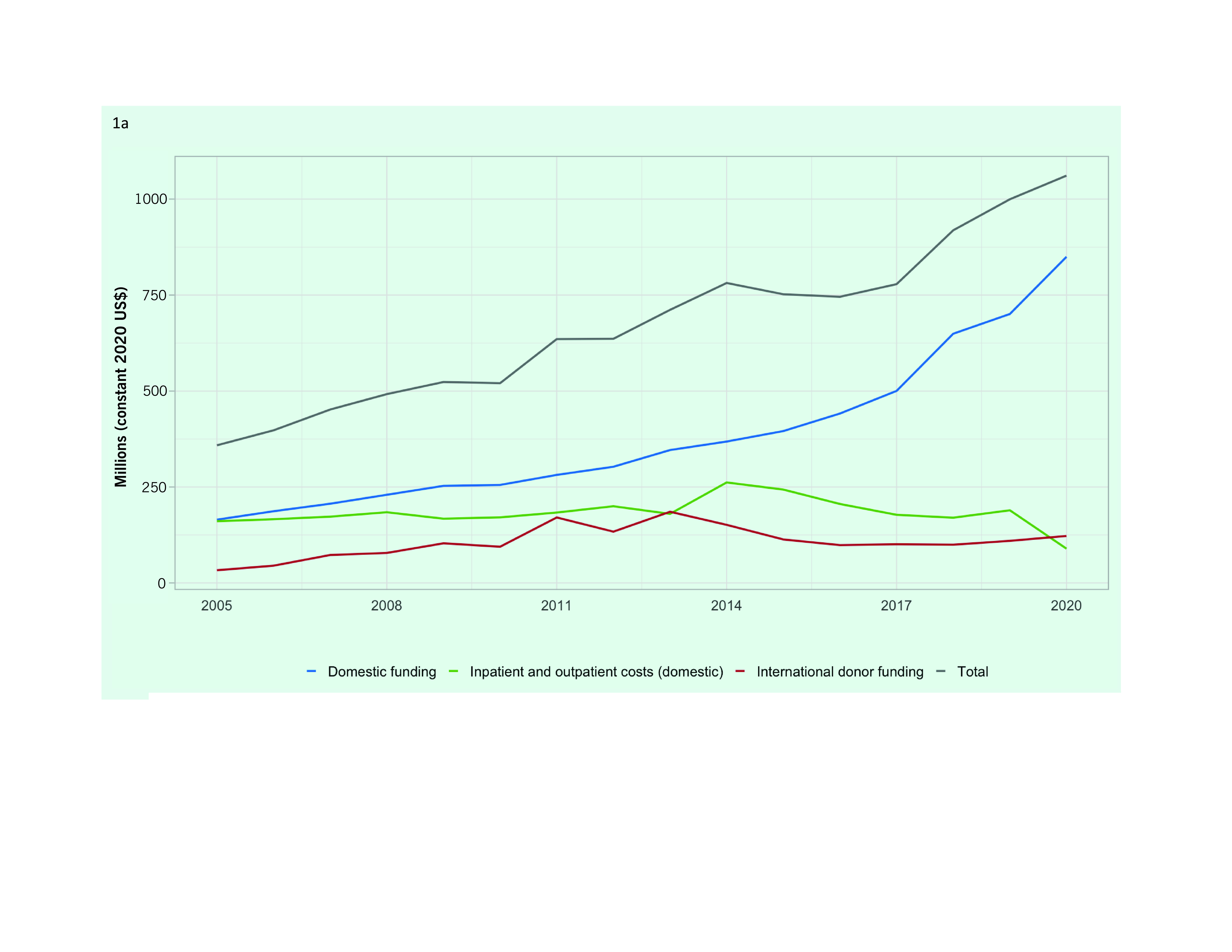
Funding for TB prevention, diagnostic and treatment services in 18 low- and middle-income countries in the Western Pacific Region, 2005–2020: (a) Total funding (US$ millions)

[**insert** Figure 1a]

Domestic funding as a percentage of total funding received for TB declined during the 2000s, dropping to its lowest levels between 2011 and 2013, but since then has increased to similar levels to those seen in the mid-2000s (**Fig. 1B**). By 2020, domestic sources accounted for 88.5% of the total TB funding among the 18 LMICs (US$ 939 of the US$ 1061 million).

Averaged across the 18 LMICs and the period 2005–2020, drug-susceptible TB (DS-TB) programme management costs accounted for the largest proportion (37%) of total TB expenditures, followed by staff costs (25%), DS-TB drugs (11%) and drug-resistant TB (DR-TB) drugs (9%). Spending by category of expenditure for each year between 2005 and 2020 is shown in **Fig. 2**.

**Fig. 1b:**
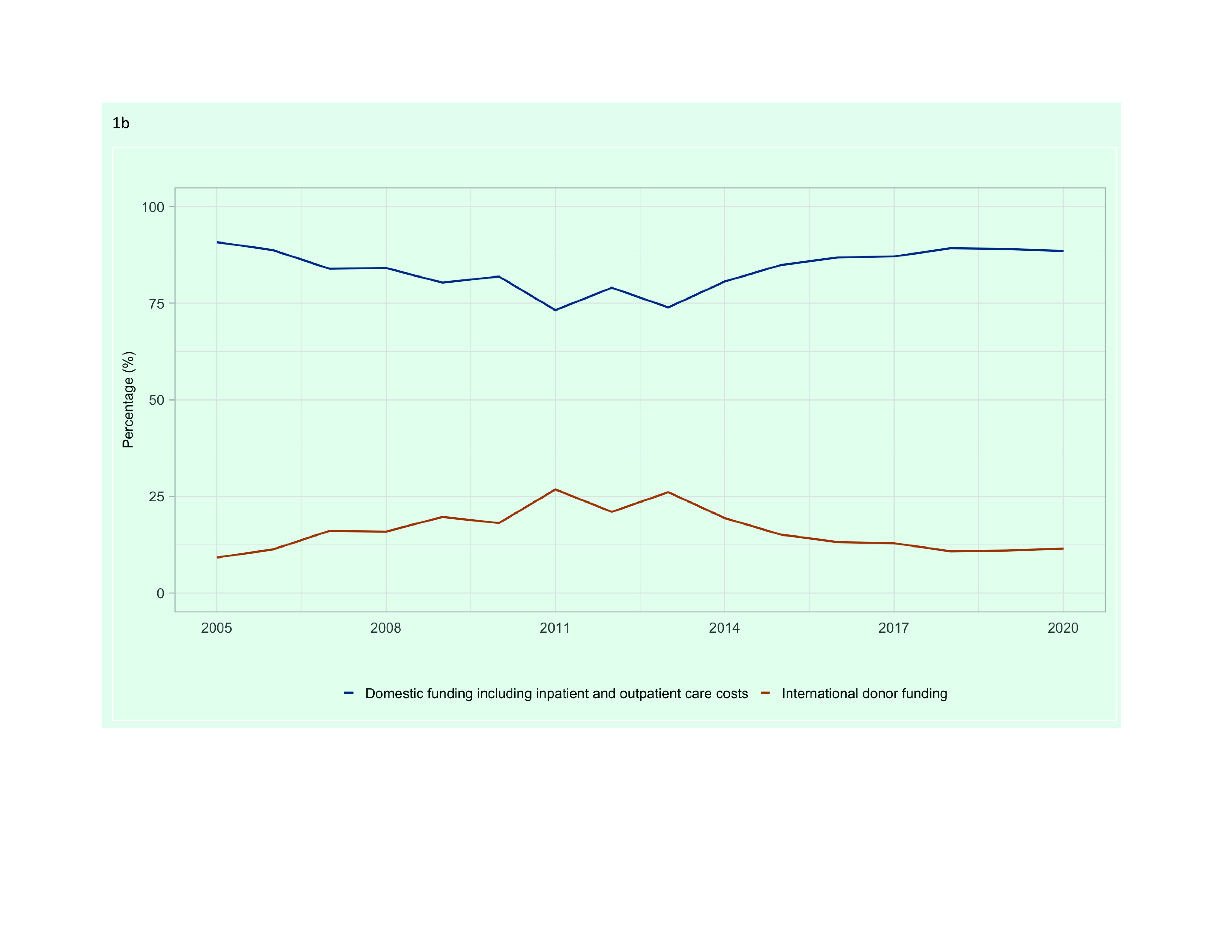
Funding for TB prevention, diagnostic and treatment services in 18 low- and middle-income countries in the Western Pacific Region, 2005–2020: (b) Domestic and international donor funding as a percentage of total funding (%)

[**insert** Figure 1b]

**Fig. 2:**
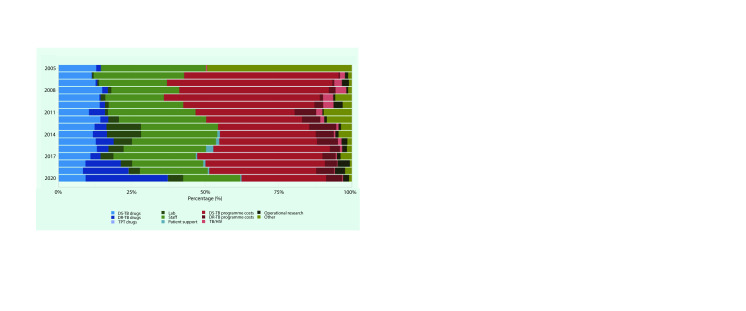
Average spending by category of expenditurea (as reported in national TB strategic plans) by 18 low- and middle-income countries in the Western Pacific Region, 2005–2020 (% of total)

[**insert** Figure 2]

### TB financing patterns in seven regional priority countries

**Fig. 3:**
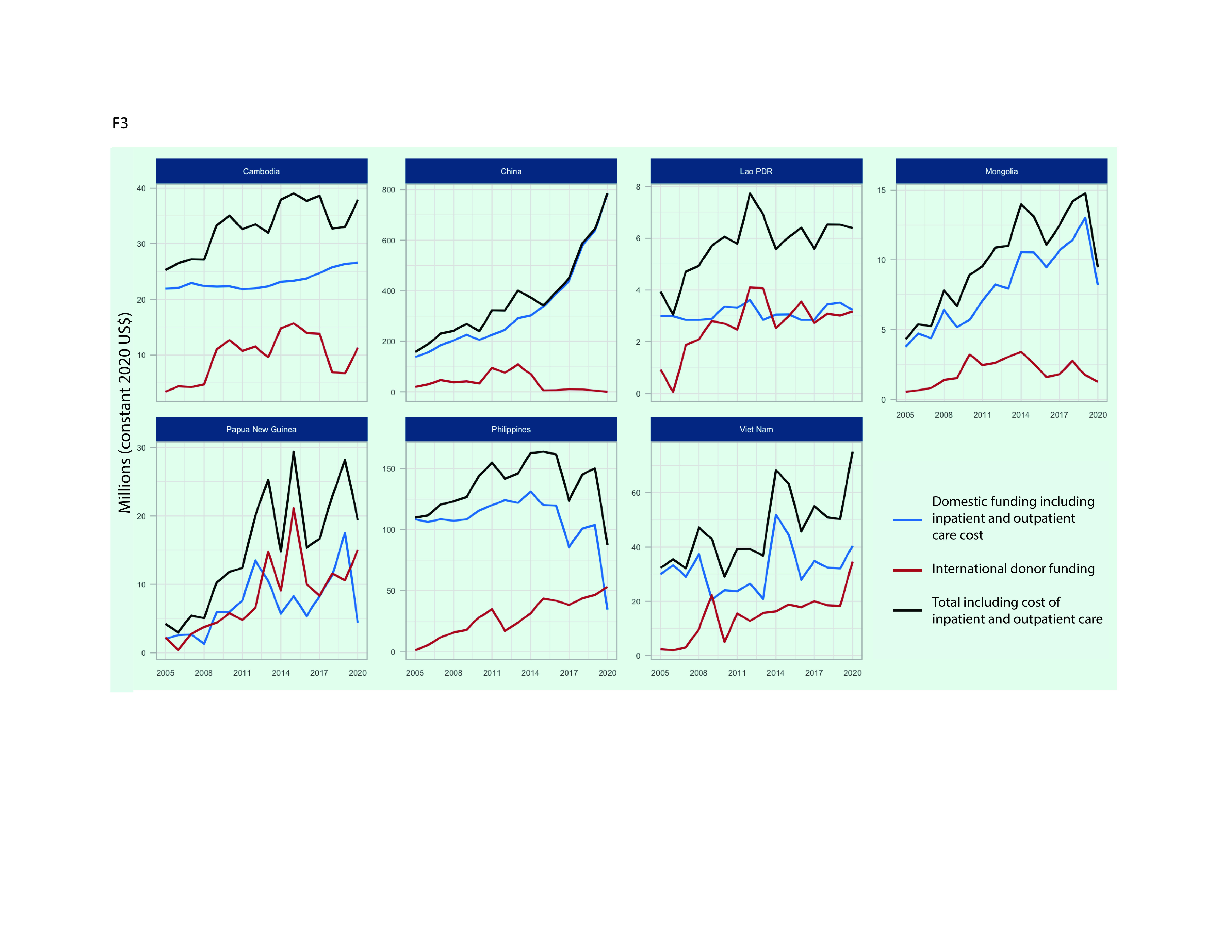
Funding for TB prevention, diagnostic and treatment services in seven priority countries in the Western Pacific Region, total amount and by funding source, 2005–2020 (US$ millions)

Funding for TB prevention, diagnostic and treatment services in the seven priority countries increased from US$ 340 million in 2005 to US$ 658 million in 2015 and US$ 1020 million in 2020. In 2020, China alone accounted for 77% of the total amount of available funding for TB in the 18 LMICs in the Western Pacific Region (US$ 1.06 billion), with a quintupling of its (mostly domestic) funding for TB between 2005 and 2020 (**Fig. 3**). Funding the TB response in the other six countries increased from US$ 180 million in 2005 to US$ 315 million in 2015, before falling back to US$ 236 million in 2020. Large increases in the total funding available for TB were observed in the late 2000s in the Lao People's Democratic Republic and in the Philippines, and in the 2010s in Viet Nam. Cambodia mobilized substantial international funding in the late 2000s and early 2010s, while Mongolia increased both its domestic and international allocations for TB response between 2008 and 2012 (**Fig. 3**).

[**insert** Figure 3]

In 2020, received funding as a percentage of the total budget required averaged 70% in the seven priority countries (US$ 952 million of US$ 1.36 billion), with large differences across countries ranging from 37% to 158% (**Table 1**). Expenditure as a proportion of received funding averaged 99% (US$ 940 million of US$ 952 million), and in no country was lower than 84%.

**Table 1:** Budgets, expenditure and received funding for TB prevention, diagnostic and treatment services reported by seven countries in the Western Pacific Region, total and proportion available and implementation rates, 2020 (current US$ millions)

Country	Funding			Total received funding as a proportion of total budget required (B)/(A) × 100	Total expenditure as a proportion of received funding (C)/(B) × 100
	Total budget required (A)	Total received funding (B)	Total expenditure (C)		
Cambodia	33	17	14	51%	86%
China	994	785	785	79%	100%
Lao People's Democratic Republic PDR	2	4	3	158%	84%
Mongolia	6	5	5	95%	100%
Papua New Guinea	34	17	17	50%	100%
Philippines	217	81	80	37%	100%
Viet Nam	71	43	36	60%	85%
**Total**	1357	952	940	70%	99%

Lao People's Democratic Republic PDR: Lao People's Democratic Republic.

*Source*: Global tuberculosis databases. (*4*)

In 2020, domestic funding for TB across the seven priority countries totalled US$ 902 million or 88% of the total investment in TB responses (US$ 1.02 billion), with China fully funding domestically (**Fig. 3**), while the other six countries funded on average 54% of the total cost of their programmes (**Table 2**). Cambodia’s domestic funding for TB increased from US$ 21.0 million.

**Table 2:** Funding for TB prevention, diagnostic and treatment services, available funding from domestic and international donor sources in seven priority countries in the Western Pacific Region, 2020 (current US$ millions)

Country	Funding			Source of funding (% of total)			
	Total received funding (A)	Estimated inpatient and outpatient care costs (B)	Estimated total resources required for TB care (A+B)	Domestic funding	International donor funding		
					Global Fund	USAID	Other^a^
China	785	–	785	100%	0%	0%	0%
Cambodia	17	21	38	70%	22%	3%	5%
Lao People's Democratic Republic PDR	4	2	6	50%	50%	0%	0%
Mongolia	5	4	9	87%	11%	0%	2%
Papua New Guinea	17	2	19	22%	58%	0%	20%
Philippines	81	7	88	39%	46%	15%	0%
Viet Nam	43	32	75	54%	45%	1%	0%
**Total**	952	68	1020	88%	10%	1%	1%

Lao People's Democratic Republic PDR: Lao People's Democratic Republic; USAID: United States Agency for International Development.

^a^ Includes grants.

*Source*: Global tuberculosis databases. (*4*)

in 2004 to US$ 26.6 million in 2020 but was offset by a decline in international financing so that overall total TB funding decreased between 2017 and 2019. In contrast, in Viet Nam, domestic funding fluctuated over the period, rising from US$ 21 million in 2009 to US$ 51 million in 2014 but dropping back to US$ 40 million in 2020. This fluctuation was driven by changes in country-reported domestic funding – which fell from US$ 10.0 million in 2006 to US$ 1.1 million in 2019 before recovering to US$ 7.9 million in 2020 – as well as by increases in the estimated cost of delivering inpatient and outpatient care (from US$ 18 million in 2004 to US$ 32 million in 2020). Over the period covered by this report, the contribution of domestic to total funding in the Lao People's Democratic Republic has remained relatively low, at around 50%.

**Fig. 4:**
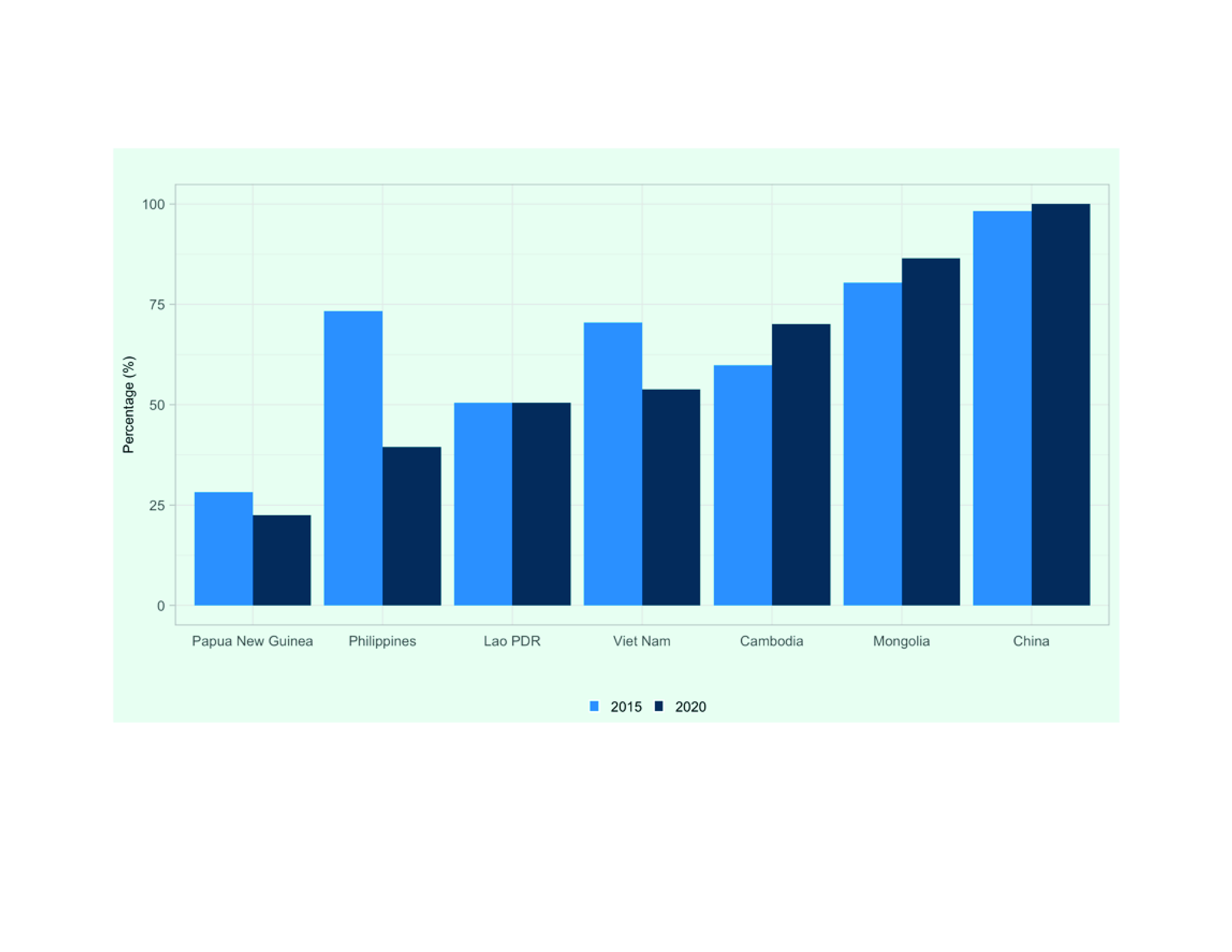
Contribution of domestic financing for TB prevention, diagnostic and treatment services in seven priority countries in the Western Pacific Region, 2015a and 2020 (as % of total received funding)

Domestic funding contributions in 2020 compared with those in 2015 show that the share of domestic funding available to meet total resources required by country NSPs decreased in Papua New Guinea (from 28% to 22%), the Philippines (from 73% to 40%) and Viet Nam (from 70% to 54%) but increased in China (from 98% to 100%), Cambodia (from 60% to 70%) and Mongolia (from 80% to 86%) and remained unchanged in the Lao People's Democratic Republic (50%) (**Fig. 4**).

[**insert** Figure 4]

**Fig. 5:**
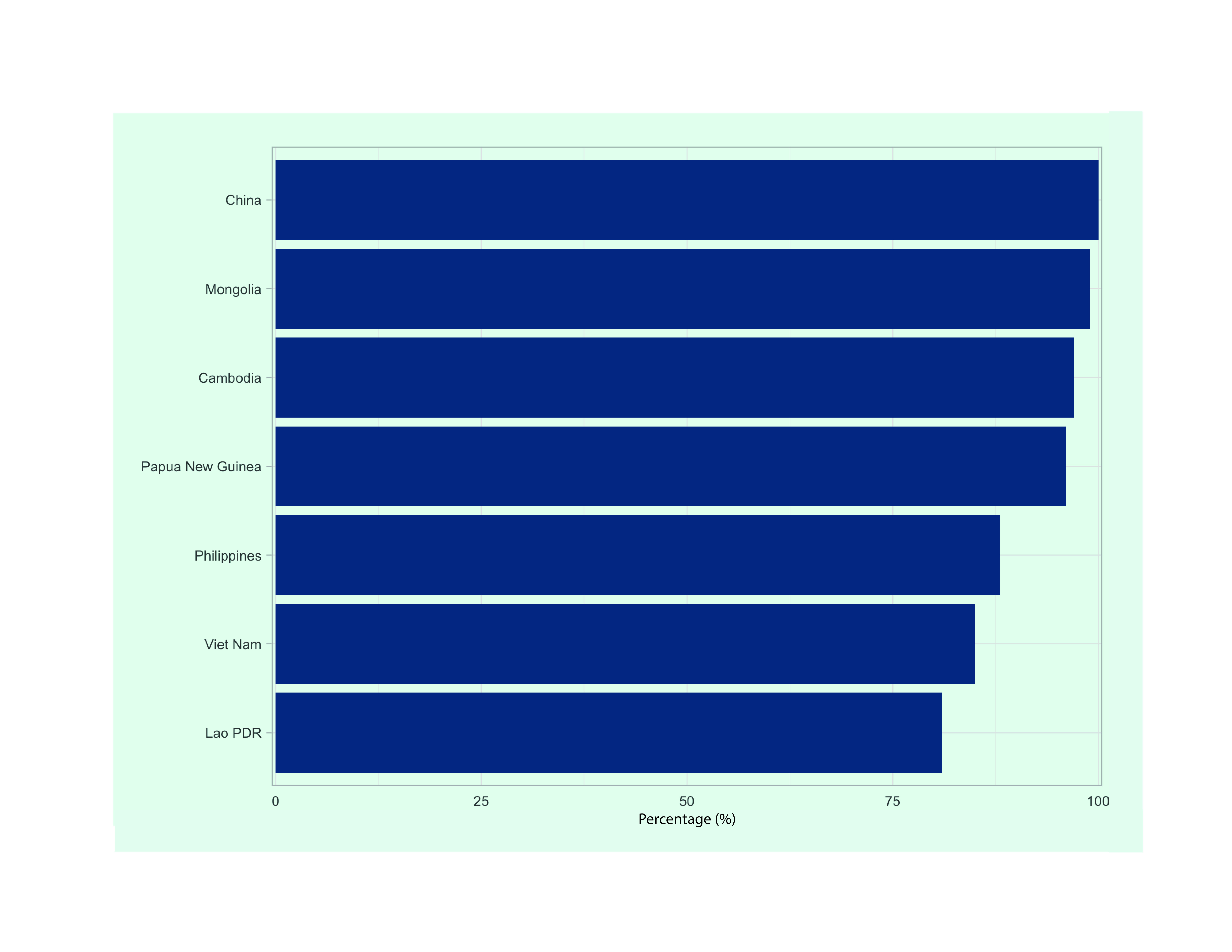
Implementation ratesa of domestic and international funding allocated for TB prevention, diagnostic and treatment services in seven priority countries in the Western Pacific Region, 2015–2020

Annual average implementation rates of domestic and international funding allocated for the TB response in the seven priority countries over the 2015–2020 period ranged from 81% in the Lao People's Democratic Republic to 100% in China (**Fig. 5**).

[**insert** Figure 5]

**Fig. 6:**
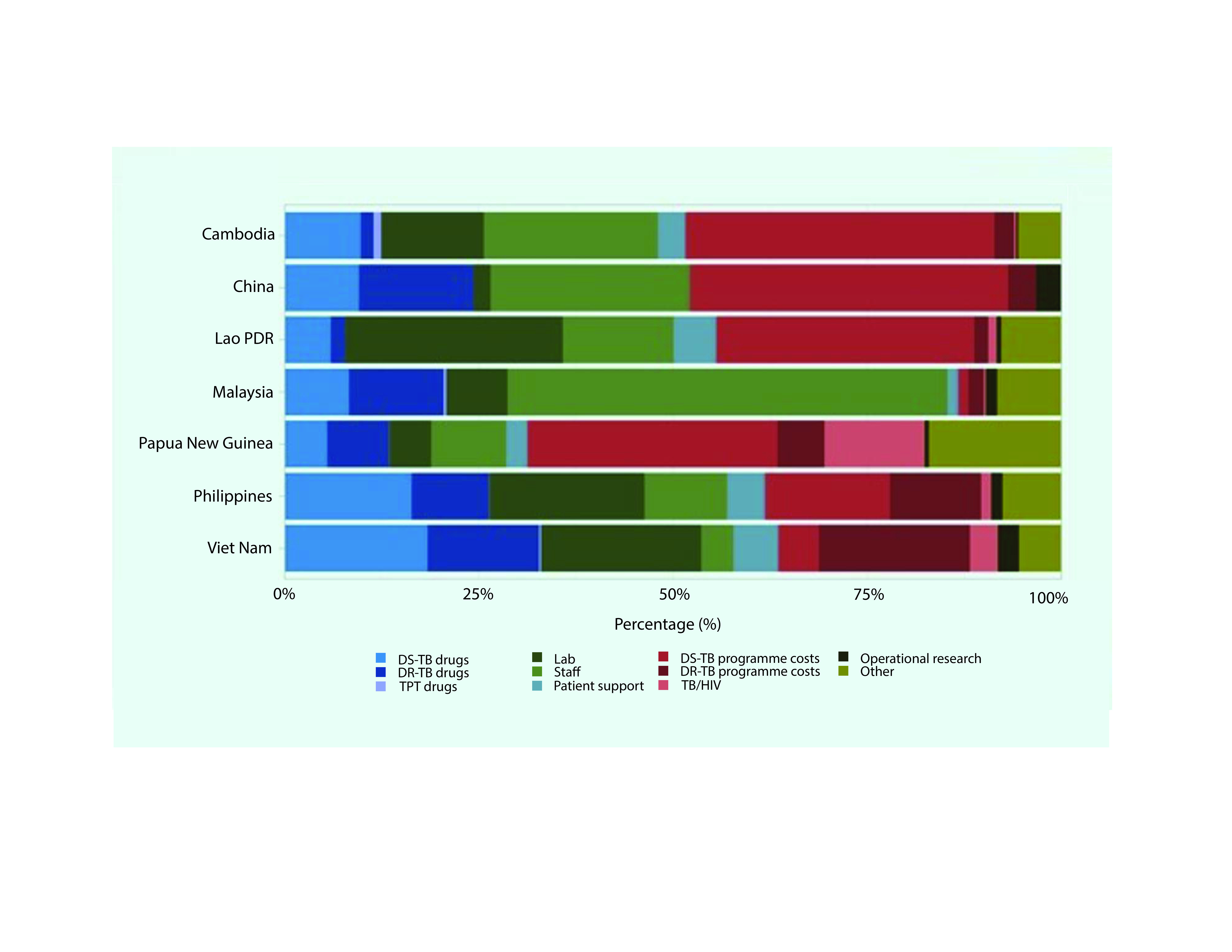
Spending on TB prevention, diagnostic and treatment servicesa by expenditure category in seven priority countries in the Western Pacific Region, 2020

In 2020, inpatient and outpatient care costs in the six priority countries excluding China were estimated to be US$ 68 million (**Table 2**). The remaining US$ 952 million of the total amount of received funding for TB in the seven priority countries in 2020

(US$ 1020 million) derives from reported TB programme spending. In most countries, programme costs for DS-TB or DR-TB specific activities accounted for the largest share of TB programme expenditures (**Fig. 6**), together averaging 31% across the seven countries, with the highest percentage reported at 45% in China, followed by Cambodia (42%), Papua New Guinea (38%) and the Lao People's Democratic Republic (35%). First-line and second-line treatment drugs combined represented 33% of the total reported programme expenditure in Viet Nam, 26% in the Philippines and 24% in China (**Fig. 6**). The proportion of spending for collaborative TB/HIV activities ranged from 13% in Papua New Guinea to 4% in Viet Nam. The proportion of total TB spending for patient support ranged from 0% to 6%, for operational research from 0% to 3% and for TB preventive treatment (TPT) from 0% to 1%.

[**insert** Figure 6]

### Reported and estimated funding gaps in the regional priority countries

**Fig. 7:**
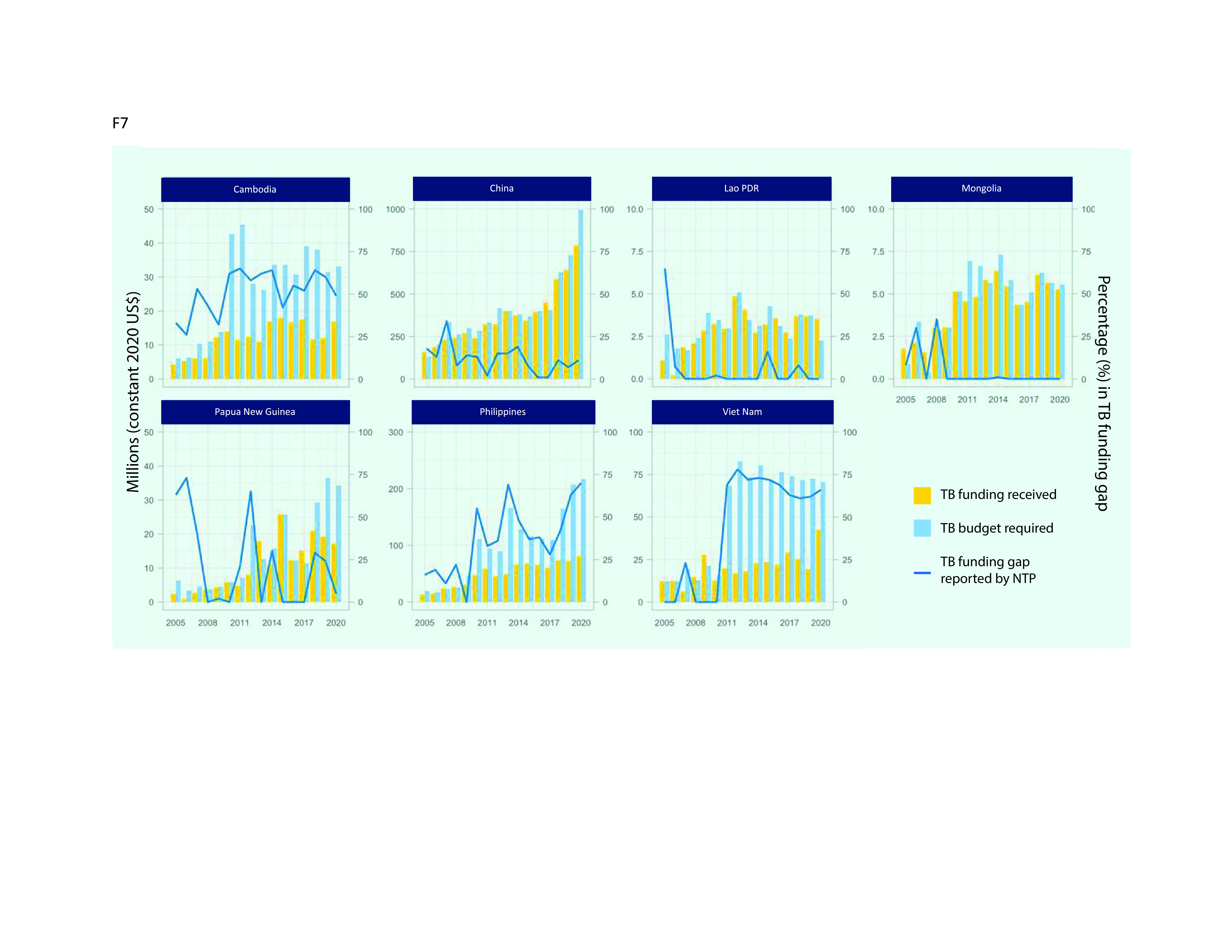
TB funding received, budgets required for national strategic plans and NTP-reported funding gapsa in seven priority countries in the Western Pacific Region, 2005–2020

TB funding gaps, reported by NTPs (and calculated as NSP budget requirements as a percentage of the expected amount of funding available from domestic or donor sources), have varied substantially not only within individual countries during 2005–2020 but also between the seven priority countries, as shown by the solid line in **Fig. 7**. Large funding gaps were consistently reported by Cambodia (ranging from 42% to 49%), the Philippines (from 37% to 70%) and Viet Nam (from 66% to 72%); in contrast, the Lao People's Democratic Republic and Mongolia reported only small or no funding gaps during most of the period 2005–2020. Fluctuating funding gaps were observed in China (from 0% to 34%) and most noticeably in Papua New Guinea (from 0% to 73%). The actual funding received exceeded the original forecast budget requirements in some years in some countries, including in China (in 2005, 2013 and 2017), the Lao People's Democratic Republic (in 2007, 2016, 2017 and 2020), Mongolia (2005, 2007–2009, 2013 and 2020) and Papua New Guinea (2013), pointing to successful funding mobilization periods in the midst of persistent potential funding gaps.

**Fig. 8:**
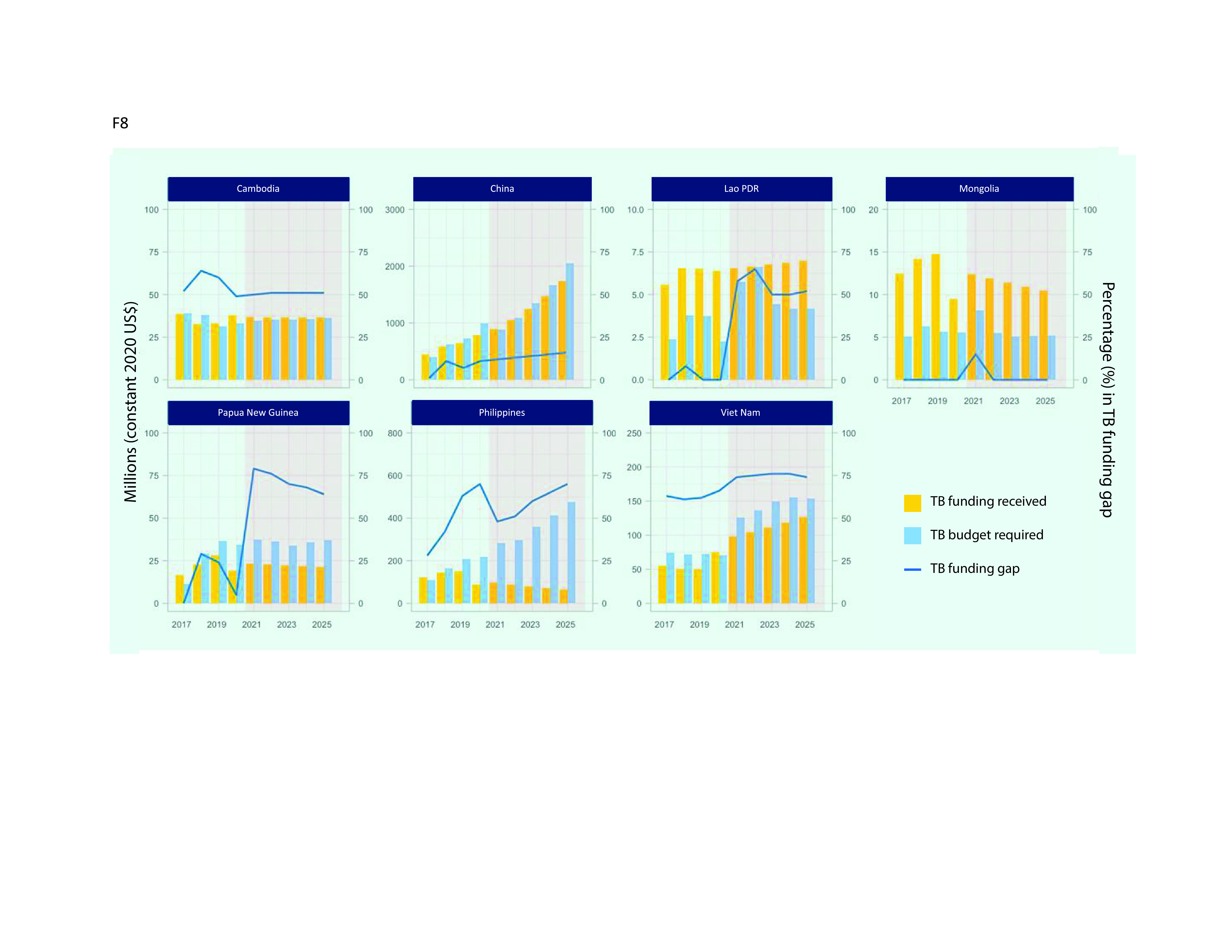
TB funding received, budgets required for national strategic plans and NTP-reported funding gapsa in seven priority countries in the Western Pacific Region, 2017–2025

TB funding requirements for the seven priority countries are projected to be around US$ 2.8 billion by 2025. With available funding assumed to double, from US$ 1.02 billion in 2020 to US$ 2 billion in 2025, this translates into an anticipated funding gap of US$ 830 million in 2025. Given that the funding gap in 2020 was estimated to be US$ 326 million, this represents a considerable widening of the funding gap over the period 2020–2025, from 24% to 30%. The average projections mask considerable variation between countries; between 2020 and 2025, funding gaps are projected to increase from 49% to 51% in Cambodia, from 11% to 16% in China, from 0% to 52% in the Lao People's Democratic Republic, from 5% to 64% in Papua New Guinea and from 66% to 74% in Viet Nam (**Fig. 8**).

[**insert** Figure 7]

[**insert** Figure 8]

### Domestic funding for TB during the COVID-19 pandemic

Five countries in the Western Pacific Region, including four priority countries, reported that part of their NTP budget was re-allocated to the COVID-19 response in early 2020. In the Philippines, the national budget allocated for NTP declined by 45%, from US$ 19 million in 2020 to US$ 10 million in 2021.

## Discussion

Total spending on TB prevention, diagnostic and treatment services in the seven priority countries in the Western Pacific Region increased by nearly 60%, from US$ 658 million in 2015 to US$ 1020 million in 2020. This was mostly driven by increases in the amount of available domestic funding, which rose from US$ 547 million in 2015 to US$ 902 million in 2020. Much of this recent surge in domestic funding has occurred in China, which alone accounted for almost three quarters (74%) of the total amount of available domestic funding in 2020 (US$ 902 million). Among the other six priority countries, including the Lao People's Democratic Republic, Papua New Guinea, the Philippines and Viet Nam, the level of domestic funding (as a proportion of total funding) has remained largely unchanged, and these countries remain reliant on donor funding. For these seven priority countries, it is anticipated that the funding gap will reach 30% by 2025, up from 24% in 2020. This projected further widening of the funding gap stems from the assumption that domestic and international funding availability remains unchanged while resource requirements to meet country TB targets will increase. Although data are limited at present, evidence suggests that the COVID-19 pandemic has impacted the allocation of funding for TB prevention, diagnostic and treatment services in 2020 across the Region.

Despite the progress made by countries to mobilize funding for TB prevention, diagnosis and treatment, at US$ 1061 million, available funding in 2020 was just 44% of the annual target set for LMICs in the Western Pacific Region, (*8*) indicating an urgent need for more investment. While the observed increase in domestic funding in the 18 LMICs and the seven priority countries overall reflects a firm political commitment to ending TB in the Region, further domestic funding increases are critical, especially in countries that rely on external funding such as in Cambodia, the Lao People's Democratic Republic, Papua New Guinea, the Philippines and Viet Nam. This is particularly important given that steady economic growth across all LMICs in the Region has meant that sources of external funding are generally declining, placing a greater onus on domestic funding to maintain essential public health functions for TB prevention, diagnosis and treatment. (*9*) Moreover, external funding is often tied to specific TB intervention areas and cannot be used to fund core programme staff or TB care delivery at the facility level. Implementation of all received funding has been challenging in a few countries that are still reliant on donor funding (e.g. the Lao People's Democratic Republic, the Philippines and Viet Nam).

Analysis of TB investments by intervention area showed that TB programme priorities have changed over time. A rise in the number of DR-TB cases enrolled in care (these increased by 70% in the Region from 2015 to 2020) (*4*) are likely to be behind recent allocation increases for second-line drugs regionally and, in particular, in countries with high multidrug-resistant TB (MDR-TB) burdens (China, Mongolia, Papua New Guinea, the Philippines and Viet Nam). Given that increases in funding for second-line drugs is often at the expense of other programme components, global efforts to reduce prices of DR-TB drugs and expand the use of shorter MDR-TB regimens remain essential.

Allocation of TB funding to TPT, patient support and operational research remains low, collectively accounting for less than 6% of total NSP spending in most countries. Despite commitments made by world leaders at the 2018 UNHLM to treat 30 million people with TB infection during 2018–2022, by the end of 2020, only 8.7 million people had received TPT. (*1*) Increased funding for this intervention is thus essential if TPT targets are to be met. Patient support interventions aimed at reducing barriers to accessing TB services such as in-kind, vouchers, cash allowances and nutrition support are also widely seen as increasingly important, especially in countries where high proportions of TB-affected families face catastrophic costs. Over one third (34%) of TB-affected families in Papua New Guinea are unable to afford the cost of TB treatment; this proportion rises to 92% in Solomon Islands. (*1*) Efforts are also needed to increase social protection measures for TB-affected families, interventions which often fall outside health or TB budgets. While TB research outputs in the Region as a whole increased by 8.8% annually between 2000 and 2018, there are still major knowledge gaps in some countries such as the Lao People's Democratic Republic and Mongolia. (*10*) Operational research can generate context-specific evidence to inform decision-making and improvements to national programmes, and thus requires adequate funding.

Projected increases in TB investment in the seven priority countries (from US$ 1.02 billion in 2020 to US$ 2 billion in 2025) are likely to be insufficient to mitigate existing funding gaps. Instead, gaps are projected to increase further, from US$ 326 million in 2020 to US$ 830 million in 2025. While the competing priorities of the COVID-19 response may impact these funding gaps in the short-term, health system-wide improvements made during the pandemic may, in the longer term, enhance TB prevention, diagnosis and treatment by facilitating accelerated uptake of digital tools and collaboration between disease programmes and sectors, (*11*) increasing efficiencies, and expanding use of personalized and remote patient support. (*12*) The pandemic has also highlighted the importance of sufficiently funded and resilient health systems, (*13*) not just for COVID-19 response but for all infectious disease control and prevention services.

This descriptive analysis has several limitations. First, WHO TB financing data for LMICs from 2003 to 2020 were not complete, especially in the early 2000s when global reporting of TB financing data was relatively new. Therefore, the observed patterns may not have accurately captured the real situation for the early reporting years. Second, unlike recent financial modelling studies, (*14*) WHO estimates of government and international funding for TB only included public spending (i.e. private spending was not included); moreover, no additional imputations (beyond what is already included in WHO TB finance data) were made for missing data (by intervention) or for out-of-pocket and private spending. Funding of TB services by local governments outside of the national TB budget and by other health programmes through integrated approaches was also not included. Third, differences in the categorization of budgets and funding streams between countries are likely to lead to under and overestimation of funding proportions by category. For example, drugs for TPT may be reported as DS-TB drugs in some countries. Fourthly, the assumptions used to project available funding and to impute missing forecasts by country-year were made by the authors without consultation with individual countries. Possible future changes in the costs of drugs and diagnostics were also not considered, and therefore the calculated funding gap may also be an under or overestimate.

Despite these limitations, this report provides an overview of domestic and international spending on TB prevention, diagnostic and treatment services in the Western Pacific Region between 2005 and 2020. It also provides a more detailed, short-term (2015–2020) assessment of TB funding and expenditure in seven priority countries that collectively account for 85% of the Region’s notified cases. Periodic analysis and reporting of TB funding, budget and expenditure data remain essential not only to monitor TB financing, identify major funding gaps and inform corrective actions, but also to advocate for government commitment to the goal of ending TB. (*15*) This analysis clearly shows that if the Western Pacific Region is to achieve its End TB Strategy targets, additional funding needs to be mobilized. The economic case for doing so is strong. Economic modelling from four high-TB burden countries in the Region has demonstrated that TB care and prevention is an extremely profitable investment that provides a multifold return on investment. (*16*) An evaluation of the economic impact of TB mortality in 120 LMICs, including the 18 LMICs in the Western Pacific Region, estimated that the cost of not investing enough in TB care to meet the 2030 targets would result in 31.8 million (95% confidence interval [CI], 25.2–39.5 million) deaths, which corresponds to an economic loss of US$ 17.5 trillion (95% CI: 14.9–20.4 trillion) during 2020–2050 globally. (*17*) The *Western Pacific Regional Framework to End TB: 2021–2030*, endorsed by Member States in October 2021, reiterated the importance of securing sustainable and adequate financing for TB programmes through a whole-of-government and multisectoral approach. (*2*) Securing high-level political commitment, ensuring coordination across sectors, and shaping a national response to TB as part of the drive towards the goal of universal health coverage will be key to advancing policy dialogue to achieve sustainable and adequate financing for TB.
